# Bringing Light to Transcription: The Optogenetics Repertoire

**DOI:** 10.3389/fgene.2018.00518

**Published:** 2018-11-02

**Authors:** Lorena de Mena, Patrick Rizk, Diego E. Rincon-Limas

**Affiliations:** ^1^Department of Neurology, McKnight Brain Institute, University of Florida, Gainesville, FL, United States; ^2^Department of Neuroscience, Genetics Institute, Center for Translational Research in Neurodegenerative Disease, University of Florida, Gainesville, FL, United States

**Keywords:** optogenetics, phytochrome B, cryptochrome, LOV, UVR8, light, transcription, gene expression

## Abstract

The ability to manipulate expression of exogenous genes in particular regions of living organisms has profoundly transformed the way we study biomolecular processes involved in both normal development and disease. Unfortunately, most of the classical inducible systems lack fine spatial and temporal accuracy, thereby limiting the study of molecular events that strongly depend on time, duration of activation, or cellular localization. By exploiting genetically engineered photo sensing proteins that respond to specific wavelengths, we can now provide acute control of numerous molecular activities with unprecedented precision. In this review, we present a comprehensive breakdown of all of the current optogenetic systems adapted to regulate gene expression in both unicellular and multicellular organisms. We focus on the advantages and disadvantages of these different tools and discuss current and future challenges in the successful translation to more complex organisms.

## Introduction

The ability to artificially trigger gene transcription using external stimuli allows for the control of the synthesis of proteins involved in a variety of cellular processes such as cell proliferation, differentiation and death. Initially, engineered expression systems capitalized on enhancer or promoter sequences that included binding sites of distinct transcription factors (TF), usually derived from viruses (CMV from cytomegalovirus), prokaryotes (LexAop-LexA from *Escherichia coli*), yeast (UAS-Gal4 from *Saccharomyces cerevisiae*), or artificially synthesized promoters. Thus, upon addition of tissue-specific sequences, such systems allowed manipulation of exogenous genes in particular regions of living organisms (Fischer et al., 1988; Byrne and Ruddle, 1989; Yarranton, 1992; Brand and Perrimon, 1993; Sun et al., 2007). Still, these tools were limited to permanent gene activation. In an attempt to attain temporal control, further techniques exploited the use of small chemical compounds that targeted particular chemical sensors or engineered proteins (Picard et al., 1988; Tanguay, 1988; Gossen and Bujard, 1992; No et al., 1996; Osterwalder et al., 2001). These methods proved to be instrumental in explaining a great variety of cellular networks and processes. Yet, side effects and the reduced accuracy associated with chemical induction gave way for the introduction of more precise approaches.

The recent emergence of optogenetics opened an exciting new door for finer spatial and temporal regulation of cellular activity (Fenno et al., 2011). Optogenetics alludes to the manipulation of natural photo switches, which respond to specific wavelengths rapidly and precisely. Because light acts as a catalyst, the delivery of the stimulus is immediate, easily controlled over time, target specific, and rapidly reversed with little to no unexpected toxic effects. Thus, it is not surprising that in the past 15 years, the toolkit of engineered photo-responsive proteins has quickly expanded from light dependent channels aimed at interrogating neural networks, to non-channel proteins designed to induce protein-protein interactions, manipulate enzyme activity, control subcellular localization, and regulate gene transcription to name a few (Deisseroth, 2011).

In this review we will address the most recent advances in the design and utilization of optogenetic tools to control gene transcription in both unicellular and multicellular organisms. We will focus on the advantages and disadvantages of the different tools described in the literature and will discuss current and future challenges encountered in their translation to living organisms.

## A Photon of Options

In nature, photoreceptors function as detectors of optical stimuli that allow the carrier organism to quickly respond to the environment. These sensor-proteins undergo a conformational change via absorption of a photon by a small cofactor, chromophore, tightly bound to the protein. Meanwhile, removal of the light stimuli provokes the photoreceptor to return to its original conformation. This return to the ground state can take from seconds to hours (Pathak et al., 2013). Different photoreceptors react differently to the light-dependent conformational changes. In some cases, activation leads to a direct response of the effector domain. In other cases, activation favors homodimerization or heterodimerization with particular binding partners, to ultimately trigger the response.

Currently, photoreceptors utilized in the generation of optogenetic tools can be divided according to three main chromophores: phycocyanobilin (PCB), flavin adenine dinucleotide (FAD) and flavin mononucleotide (FMN). PCB is a blue tetrapyrrole chromophore from cyanobacteria that acts as light energy acceptor in phycobiliproteins (Bhoo et al., 2001; Lamparter et al., 2004; Rockwell et al., 2006). FAD is a flavin conjugated with an adenosine diphosphate derived from riboflavins and associated with blue-dependent photoproteins (Sancar, 1994; Lin et al., 1995). FMN, another blue light-absorbing chromophore also derived from riboflavins, is responsible for the conformational change occurring in phototropins, LOV-domains containing photo sensors (Salomon et al., 2000; Christie et al., 2012). Interestingly, there is a fourth category comprised of a family of photoreceptors reactive to ultraviolet light whose function is independent of a chromophore (Christie et al., 2012). Here, we will focus mainly on the PCB-dependent phytochrome PhyB (Anders and Essen, 2015), FDA-dependent cryptochromes (CRY2) (Lin and Shalitin, 2003), FMN-dependent LOV domains (Losi and Gartner, 2008), and UVR8 (Crefcoeur et al., 2013) photoreceptors because they are the most widely used for optical control of gene transcription (Table 1).

**Table 1:** Overview of optogenetic tools used for regulation of transcription.

Light	Chromo phore	System	Tool type	Photo sensor	Cofactor	TF	Activation wavelength	Intensity (μmol m^−2^ s^−1^)^∗^	Model	Reference
RED	PCB	PhyB-Pif	Two-hybrid	PhyB	Pif3	Gal4 DBD – Gal4 AD	660 nm/750 nm	1 or 40	Yeast	Shimizu-Sato et al., 2002
			Two-hybrid	PhyB	Pif6	TetR DBD-VP16 AD	660 nm/740nm	8/80	CHO-K1 cells Chicken embryos	Muller et al., 2013a,b
			Two-hybrid	PhyB	Pif6	TetR DBD-VP16 AD	660 nm/740 nm	20/20	NIH/3T3 cells Zebrafish	Beyer et al., 2015 Gomez et al., 2015
BLUE	FDA	CRY2-CIB	Two-hybrid	Cry2	CIB1	Gal4 DBD – Gal4 AD	488 nm	25 μW, 1.7 mW, 4.5 mW	HEK293 cells	Kennedy et al., 2010
			Two-hybrid	Cry2	CIB1	LexA DBD-AD Gal4	474 nm	2.5 mW cm-2	S2 cells Drosophila	Chan et al., 2015
			Heterodimer	Cry2	CIB1	*NA*^−^	Blue	42–120 mmol m^−2^ s^−1^	Zebrafish	Liu et al., 2012
		CRY2	*NA*		*NA*	Gal4 DB (1–65)-VP16AD	461 nm	7.4 mW cm^−2^	Hek293 cells Zebrafish	Pathak et al., 2017
		LITEs	Heterodimer	Cry2	CIB1	TALE – VP64 AD	473 nm	5 mW	Neuro2A cells	Konermann et al., 2013
		LACE	Heterodimer	Cry2	CIBN	VP64 AD-dCas9	450 nm	48 lumens	HEK293 cells	Polstein and Gersbach, 2015
										
	FMN	LightOn	Homodimer	VVD	*NA^+^*	Gal4(1–65 aa)	460 nm	0.84 W m^−2^ 90 mW cm^−2^	HEK293 cells Mice	Wang et al., 2012
		LITEZ	Two-hybrid	GI	FKF1	ZFP DBD-VP16 AD	450 nm	48 Lumens	HEK293T, NIH 3T3, HeLa cells	Polstein and Gersbach, 2012
		TULIP	Two-hybrid	LOV-pep	ePDZ	Gal4 DBD – Gal4 AD	461 nm	5.8 mW cm^−2^	Yeast	Pathak et al., 2014
		EL222	Dimer	LOV	*NA*	HTH DBD-VP16 AD	465 nm	8 W m^−2^	HEK293 cells Zebrafish	Motta-Mena et al., 2014
		TAEL	Dimer	LOV	*NA*	HTH DBD-KalTA4AD	488 nm	1.6 mW cm^−2^	HEK293 cells Zebrafish	Reade et al., 2017
		LANS	NLS shuttle	asLOV2	*NA*	LexA DBD-Gal4 AD	455 nm	6 mW cm^−2^	Yeast	Yumerefendi et al., 2015
		LINuS	NLS shuttle	asLOV2	*NA*	LexA DBD-VP64 AD	460 nm	10	Yeast HEK293T cells	Niopek et al., 2014
		LINX	NES shuttle	asLOV2	*NA*	LexA DBD-Gal4 AD	488 nm	8 μs/pixel	HEK293 cells	Yumerefendi et al., 2016
		LEXY	NES shuttle	asLOV2	*NA*	LexA DBD-VP64 AD	490 nm	*Not specified*	H1299 cells	Niopek et al., 2016
		BLITZ	Dimer	Cry2/asLOV2	CIBN/*NA*	TetR-VP16 AD	473 nm	1.7 mW	HEK293 cells	Lee et al., 2017a,b
UV-B	*NA*	UVR8-COP1	Two-hybrid	UVR8	COP1	Gal4 DBD-NF-κB AD	280–375 nm 290–310 nm	25 J m^−2^ 0.7 mW	U2OS cells	Rizzini et al., 2011

*^∗^Unless specified in other light units; NA, not applicable; NA^−^ light negatively regulate gene expression.*

### Running the Red Lights

#### Phytochromes

Phytochromes (Phy) are photoreceptors sensitive to red and far red light found in bacteria, cyanobacteria, fungi and plants. These photoactive proteins are responsible for several light-dependent functions such as growth, development, seed germination and flowering, as well as a wide spectrum of mechanisms regulated by circadian rhythms (Davis et al., 1999; Auldridge and Forest, 2011). Although phytochromes consist of five identified members (PhyA-E), only PhyB has been extensively studied for optogenetic purposes (Sharrock and Quail, 1989).

Phytochromes are bilin-regulated dimers consisting of a photosensory N-terminal domain (PAS domain) that interacts with a bilin chromophore; and a C-terminal domain (GAF domain) that binds to cGMP to exert its regulatory function. In order for the photoswitching to occur, a phytochrome isomerizes with a tetrapyrrole chromophore referred to as phycocyanobilin (PCB), which is endogenous to plants, but needs to be exogenously provided or genetically engineered when working in animal models (Anders and Essen, 2015). In the dark, phytochromes exist as an inactive form (Pr). Upon red light stimulation (∼630 nm), Pr undergoes a conformational change turning into the active form (Pfr) (Rockwell et al., 2006; Auldridge and Forest, 2011). Then, Pfr can heterodimerize with phytochrome-interacting factors, such as PIF3 or PIF6, to exert a variety of functions. Due to its strong conformational stability, a short pulse of red illumination can maintain the system active for hours (Muller et al., 2013a).

**FIGURE 1:**
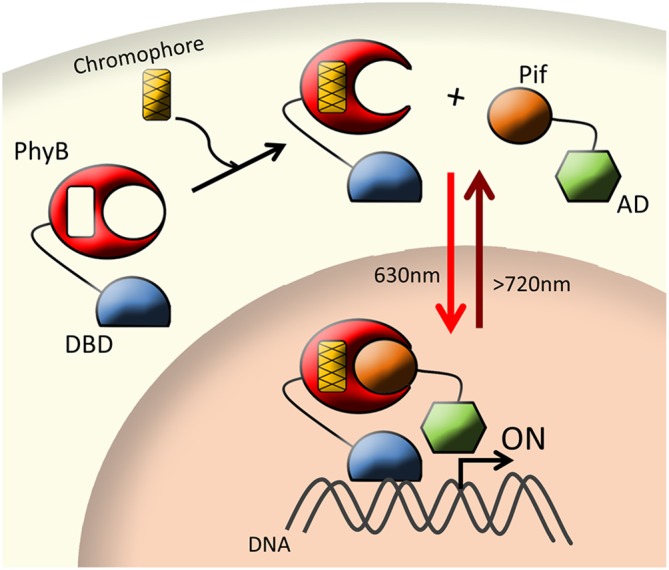
Schematic layout of red-light inducible system (PhyB-PIF). In presence of the chromophore and upon red light irradiation (∼630 nm), PhyB interacts with its binding partner PIF to reconstitute a split transcription factor (DBD and AD) and trigger gene expression. Conversely, under far-red light irradiation (>720 nm), the PhyB-PIF complex dissociates and transcription ceases. The yellow box represents the exogenous chromophore. Arrows depict direction of activity and light conditions: the red arrow represents red light-dependent activation (∼630 nm), the dark red arrow represents far-red light-dependent inactivation (>720 nm), solid black arrow represents interaction of PhyB and chromophore independent of light.

Thus, PhyB-PIF in combination with two-hybrid systems can be utilized to mediate light-dependent protein-protein interactions or reconstitute split protein domains (Figure 1). This is of particular interest for transcription systems where PhyB and PIF are fused to the DNA-binding domain (DBD) and the transcriptional activation domain (AD) halves of specific transcription factors. In this case, light irradiation brings both chimeric proteins together reconstituting its function. In addition, the PhyB-PIF system can be switched off at any time. When irradiated with far-red light (>720 nm), the Pfr form absorbs a photon, dissolves the dimerization, and returns to its original inactive Pr form within milliseconds (Shimizu-Sato et al., 2002; Levskaya et al., 2009; Muller et al., 2013a).

The first phytochrome-based optogenetic paradigm consisted of a fusion of the PhyB photosensory N-terminal domain with Gal4-DBD along with a fusion of PIF3 and GAL4-AD to drive gene expression in yeast (Shimizu-Sato et al., 2002). In medium containing PCB, light triggered 1000-fold expression of a LacZ reporter transgene with almost undetectable levels in dark. In addition, far-red illumination caused immediate deactivation of the system, returning LacZ to basal levels. The authors also observed a proportional correlation between the ratio of photo-conversion and number of photons delivered (Shimizu-Sato et al., 2002). Another compelling approach proposed PhyB-PIF3 heterodimerization as a mean to spatially control post-transcriptional events in yeast. Light-dependent reconstitution of the split form of a *S. Cerevisiae* vacuolar ATPase (VMA) intein translated into a fourfold increase in the amount of conditional splice product (Tyszkiewicz and Muir, 2008). These initial results demonstrated the unprecedented power of engineered phytochrome-based optic tools to manipulate protein expression at different stages of protein synthesis.

Interestingly, the first application of the PhyB-PIF system in animal models focused on light-dependent subcellular protein translocation (Levskaya et al., 2009). By using light to recruit PIF6 to a membrane-anchored PhyB, the authors laid the foundations necessary for further experimentation in gene expression (Levskaya et al., 2009). Based on these discoveries, Weber’s lab proposed a PhyB-PIF6 based optogenetic system to control gene transcription in animal cells (Muller et al., 2013a). Similar to previous observations in yeast, upon red light stimulation, an N- terminal fusion of PIF6 with the tetracycline repressor (TetR) heterodimerizes with the photosensory domain of PhyB fused to VP16 transactivation domain, triggering a 65-fold increase of tetO-mediated reporter expression compared with unilluminated cells (Muller et al., 2013a). Additionally, PhyB-PIF transfected cells showed dose-dependent reporter activity when cultured under increasing PCB concentrations or exposed to increasing light intensities. Moreover, the authors showed fine spatial control of activation by directing light through a photomask on a monolayer of CHO-K1 cells. These results were then validated *in vivo* by promoting expression of the 121 amino acid splice variant of human vascular endothelial growth factor hVEG in chicken embryos (Muller et al., 2013a).

More recently, Beyer et al. (2015) took a similar approach in zebrafish embryos soaked in a concentrated PCB solution. Here, a nuclear export signal (NES) fused to PhyB allowed PhyB to accumulate in the cytoplasm. Thus, only after light-dependent association with full-length PIF3, PhyB translocated to the nucleus showing maximum nuclear localization after 15 min. Accordingly, irradiation of cells with far-red light resulted in complete reversion to the dark state only after 10 min (Beyer et al., 2015). A different approach proposed the use of PhyB-PIF to manipulate gene editing through adeno-associated virus (AAV) (Gomez et al., 2015). AAVs were engineered to display PIF6 motifs on the capsid to bind an NLS-tagged PhyB. Then, modulation of the red to far-red light ratio and intensities resulted in significantly enhanced efficiency of delivery to the nucleus compared to the wild type virus. Once again, a photomask was enough to direct space-resolved gene expression patterns in HeLa cells (Gomez et al., 2015).

Altogether, PhyB-PIF photodimerization has proven to efficiently manipulate gene expression throughout a variety of models with high spatiotemporal resolution. However, it is important to remember that phytochromes require the bilin cofactor PCB to absorb the energy of a photon and undergo the necessary conformational change. While synthesis of PCB is endogenous in plants and cyanobacteria, yeast and animal cells require an exogenous supply. Fortunately, it has been widely proven that both yeast and animal cell models can passively absorb PCB when supplied in the media (Shimizu-Sato et al., 2002; Levskaya et al., 2009; Toettcher et al., 2011; Muller et al., 2013a; Beyer et al., 2015). PCB can be easily extracted in the lab from *Spirulina* (Toettcher et al., 2011); or, if preferred, quality PCB extracts are available from a variety of companies at affordable prices. However, administration of PCB to multicellular organisms becomes more challenging. Passive absorption is difficult or highly inefficient in higher animal models, leaving injection as the most preferred approach (Beyer et al., 2015). Alternatively, it is possible to engineer cells to genetically synthesize PCB chromophore by converting the heme group, present in all animal organisms, to bilin. This artificial synthesis of PCB was initially demonstrated by engineering two enzymes, heme oxygenase (HO1) and phycocyanobilin: ferredoxin oxidoreductase (PcyA) in *E. coli* (Zhang et al., 2009). However, these results were partially replicated in mammalian cells only after directing localization of both engineered enzymes to mitochondria and knocking down a potential enzyme protease responsible for PcyA degradation (Muller et al., 2013c).

Recently, a new report offered an improved version of this strategy where HeLa cells were modified to express HO1 and PcyA with Ferredoxin (Fd) and Ferredoxin-NADP + reductase (FNR) derived from *Thermosynechococcus elongatus* BP-1 or *Synechocystis* sp. These four genes were engineered to synthesize PCB at sufficient concentrations in the mitochondria to drive detectable PhyB-PIF activation (Uda et al., 2017). Moreover, with a similar approach, Kyriakakis et al. (2018) observed that variation in rate of expression of HO1-PcyA versus Fd-FNR in mammalian cells has a direct correlation with the production levels of PCB in mitochondria, which is further limited on the cytoplasm by heme restriction. A complementary approach proposed reducing the metabolism of biliverdin by knocking down or knocking out the biliverdin reductase A, a mammalian endogenous PCB metabolic agent, resulting, therefore, in an increase of available PCB (Uda et al., 2017).

These new tools provide a promising opportunity to successfully establish PhyB-PIF systems into higher complex animal models and deep tissues. Nonetheless, there is still a need of further experimentation to address the efficacy, functionality, and potential side effects of such genetic modifications, including prolonged accumulation of artificially synthesized PCB, in higher organisms.

### Feeling Blue: To LOV and CRY

#### Cryptochromes

Cryptochromes (CRY) are photo sensory receptors that regulate several vital biological functions such as growth, development and flowering as well as the regulation of the circadian clock in plants and animals (Sancar, 2003; Lin and Todo, 2005). Of the three members of cryptochromes first described in *Arabidopsis thaliana*, CRY2 is of special interest as it has been the focus of several current optogenetic systems. Structurally, CRY2 contains a conserved photolyase homology region (PHR) that binds to the flavin adenine dinucleotide chromophore (FAD). This chromophore is a two-electron carrier that, upon blue light irradiation (450 nm), becomes reduced, triggering a conformational change (Yang et al., 2000; Sancar, 2004). This change results in the formation of homodimers or heterodimers with cryptochrome-interacting proteins, like cryptochrome-interacting basic helix-loop-helix 1 (CIB1) or CIB1 truncated version (CIBN) (Kennedy et al., 2010). Reversibility of the cycle occurs over time with the chromophore being returning to the ground state within minutes (a half-life of ∼12 min in dark) (Kennedy et al., 2010). Since FAD is present in most animal cells, cryptochrome-based systems do not require addition of exogenous chromophore to the media, which greatly simplifies adaptation of the system to live organisms.

**FIGURE 2:**
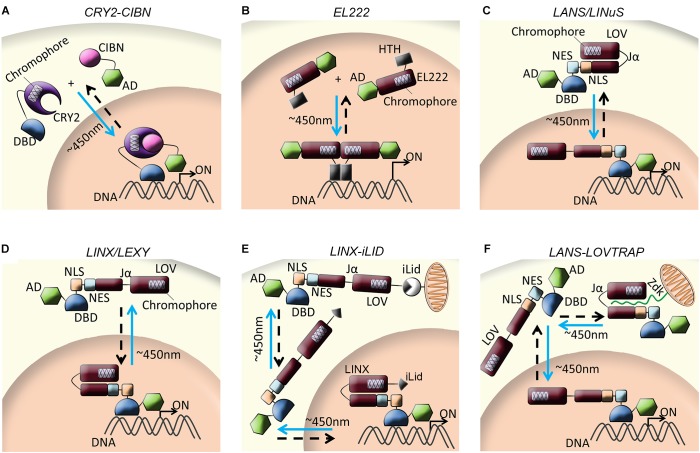
Schematic illustration of blue light-dependent optogenetic strategies. **(A)** Upon blue light irradiation (∼450 nm), Cry2 dimerizes with CIBN to induce activation of a split transcription factor (DBD and AD) and promotes gene expression. **(B)** In the presence of blue light, EL222 undocks HTH-DBD facilitating homodimerization of two EL222 proteins which, then, bind to C120 promoter to trigger transcription. **(C)** Upon blue light irradiation, a monomeric form of LOV uncages a small NLS causing the system (LANS or LINuS) to accumulate in the nucleus. In the absence of blue light or darkness, a counter-active NES peptide outside the LOV-cage allows for the system to accumulate in the cytoplasm. **(D)** In darkness, an NLS peptide outside the LOV-cage allows for the optogenetic system (LINX or LEXY) to accumulate in the nucleus and promote gene expression. After blue light irradiation, LOV uncages a small counter-active NES causing the system to translocate to the cytoplasm and transcription ceases. **(E)** In the absence of light, LINX in combination with a light dependent dimer (iLid) accumulates in the nucleus and triggers gene expression. Upon blue irradiation, LINX-iLid translocates to the cytoplasm where it dimerizes with a second iLid dimer sequestering the system to the mitochondria. **(F)** In the cytoplasm, LOV dark-state selectively binds to the peptide Zdk sequestering LOV to the mitochondria. Upon light irradiation, LOV’s conformational change allows for the release of the system from zdk and translocate to the nucleus. Arrows depict direction of activity and light conditions: blue arrow (∼450 nm) represents blue light activation, while dashed black arrow represents darkness or absence of light stimuli. For simplicity, light purple boxes inside the photo-sensing protein represent the chromophores.

Initial studies in yeast capitalized on the potential of CRY2-CIB heterodimerization to control cellular processes by light similarly to PhyB-PIF hybrid system. By combining CRY2-CIB with split Gal4, Kennedy et al. (2010) achieved strong expression of a reporter protein, Snl1, after only 4 h of blue light pulses (Figure 2A). Additionally, Liu et al. (2012) proved that CRY2-CIB dependent reporter activity in yeast cells is not only dose-dependent but also exposure-dependent. Similar results were observed in zebrafish embryos where gene expression associated with CRY2-Gal4DBD and CIB-Gal4AD fusions was only possible under blue irradiation whereas no activity was detected under red light or in darkness (Liu et al., 2012). Comparable approaches have been proposed with other split transcription factors. For instance, Chan et al. (2015) designed a light-dependent reconstitution system for split LexADBD-VP16 in *Drosophila melanogaster*. However, the unexpected high levels of transcription observed in dark required the addition of Gal80, a transcriptional repressor of Gal4, to lower the strength of the driver and consequently reduce basal expression in darkness (Chan et al., 2015).

Another interesting application resulted from the fusion of transcription activator-like effectors (TALE) to CRY2, and CIB1 fused to an effector of interest (LITEs). Here, TALE-CRY2 binds to the target gene and after blue light stimulation recruits the CIB1-effector triggering transcription (Konermann et al., 2013). Following a similar rationale, Polstein and Gersbach (2015) proposed a new system named LACE consisting of CRY2 fused with a transactivation domain and either the N- and C- terminus of the catalytically inactive dCas9 fused to CIBN. This system, when co-transfected with four gRNAs and irradiated with blue light, targeted the human IL1RN promoter, generating an 11-fold increase in mRNA within the first 2 h to a 400-fold after 30 h of light (Polstein and Gersbach, 2015). Of note, manipulation of gene editing through CRY2-CIB is also possible through a combination with a split version of Cre-recombinase (CIBN-Cre-C-terminal and CRY2-Cre-N-terminal). In transfected HEK293 cells, light dependent dimerization of Cre modules showed a dose-response increase of recombinants by 25-fold after only 15 min of light pulses and 158-fold after 24 h of continuous light pulses (Kennedy et al., 2010). Taslimi et al. (2016) furthered this research by introducing the L384F mutation in CRY2. L384F mutation extends CRY2-CIB interaction, leading to an increase in recombinase activity by 35% with a single flash of light.

Lastly, it is worth noting that CRY2-CIB heterodimerization could be also used as light-dependent transcriptional suppressor (Liu et al., 2012). CIB recognizes the CACGTG G-Box promoter, a motif identified in 5′ region of certain plant genes involved in regulation of several physiological and environmental signals (Williams et al., 1992). Thus, while in the dark, CIB is free to recognize the G-box acting as a transcription activator on a recombinant model. However, upon blue light illumination, CRY2 heterodimerizes with CIB1 suppressing its activity, therefore negatively regulating gene expression (Liu et al., 2012). A different example of CRY2 acting as a transcriptional suppressor takes advantage of a light-dependent CRY2 nuclear clearing phenomena observed in mammalian cells, and later corroborated in embryos of zebrafish (Pathak et al., 2017). In darkness, a single protein harboring CRY2, Gal4DBD, and VP16 AD (CRY-Gal-VP16) localizes in the nucleus of cells where it promotes gene expression. Then, when exposed to blue light, CRY2-tethered proteins within the nucleus cleared, resulting in a 37-fold reduction of luciferase expression levels over 24 h period (Pathak et al., 2017).

#### LOV-Domains

Since the discovery of LOV-containing proteins as regulators of phototropism in plants, LOV proteins have been described in prokaryotes, algae and fungi with a wide spectrum of roles such as kinase and phosphodiesterase functions, regulation of stress responses, cell attachment and development, as well as participation in chloroplast movements and stomatal translocation among others (Liscum and Briggs, 1995; Christie et al., 2002). The LOV domain consists of approximately 110 amino acids of a central PAS core and two alpha-helixes (A′α and Jα). Upon blue light stimulation (450–500 nm), the C4a position of the chromophore isoalloxazine ring forms a covalent thioether bond with the conserved cysteine residue within the LOV domain, unfolding the C-terminal helix. Once the light stimulation ceases, the bond hydrolyzes and the helix re-associates with the LOV domain (Woolley, 2012). This light-dependent conformational change occurs very fast, from milliseconds to minutes (Zoltowski et al., 2009). In the wild, a balance exists between dark and lit forms. However, certain direct point mutations within the LOV domain sequence can modify the kinetics of reversibility, light sensitivity and reduce spontaneous undocking in the dark (Nash et al., 2008; Zoltowski et al., 2009; Song et al., 2011). These modifications led to the design of different LOV-based optogenetic tools with different properties and kinetics. So far, current LOV-dependent photosensors are derived from four main organisms include: VVD (*Neurospora crassa* Vivid), FKF1 (*A. thaliana* Flavin-binding, kelch repeat, F-Box1), AsLOV2 (*Avena sativa* phototropin1), and EL222 (*Erythrobacter litoralis* LOV- transcription factor). They can be grouped based on their capability to either (*a*) couple allosteric regulation of the Jα-helix to induce light dependent dimerization on a two hybrid-like system, or (*b*) direct subcellular localization of the protein of interest by docking a signal peptide. These two approaches are discussed below.

##### Optical dimerization systems

One of the very first LOV-based optogenetic mechanisms to control gene transcription capitalized on the ability of *N. crassa* VVD to homodimerize. Interestingly, VVD, the smallest LOV domain–containing protein, lacks the Jα*-*helix, but possesses an N-terminal helix (Ncap) which homodimerizes upon light irradiation. Wang et al. (2012) exploited the particular properties of a mutated version of VVD (N56K and C71V double mutant) by combining it with the monomeric Gal4-DBD (1–65 aa) and the p65-AD in a two hybrid-like approach named LightON. Upon blue light stimulation, LightON triggered a 200–300-fold increase in reporter expression compared to the dark-incubated control in HEK-293 cells. In a separate experiment, LightON and mCherry reporter gene were transferred in mice through hydrodynamics-based procedure for expressing transgenes. After illumination with optical fibers, mice livers showed light-dependent spatial activation of reporter genes but only limited to 1mm from the surface. Using a similar approach, type I diabetic mice expressing insulin in a light-dependent manner presented a reduction of blood glucose compared to controls (Wang et al., 2012). However, VVD’s low affinity and long half-life (up to 48h) proved difficult to gain fine and robust control of gene expression over time. With that in mind, the same group modified the core promoter of the target gene, the number and length of UAS spacers and the amount of photoreceptor available resulting in a significant reduction of background expression of the target gene (Ma et al., 2013).

An alternative tool builds on the heterodimer proteins GI and FKF1, associated with control of flowering in *A. thaliana*. This new system, LITEZ, consists of GI fused to a zinc finger protein (ZFP), small DNA binding motifs that specifically recognized DNA triplets; and FKF1 fused to three repeats of VP16AD. By increasing the number of ZFP binding sites of the target gene, luciferase reporter showed increments in light-dependent expression between 4- and 53-fold compared to unilluminated controls in HeLa cells (Polstein and Gersbach, 2012; Wang et al., 2012). Spatiotemporal activation of GFP in cell culture was also possible by using programmable LED arrays in combination with a photomask to create specific patterns (Polstein and Gersbach, 2014). However, although LITEZ long half-life and quick activation kinetics allow gene activation with short cycles of light which reduces the risk of photo toxicity, its slow deactivation kinetics compromises its versatility and manipulation over time (Yazawa et al., 2009).

A similar strategy was created based on the light dependent heterodimerization of AsLOV2 phototropin 1 domain containing a peptide tag (LOVpep) at the C-terminus of the Jα-helix with an engineered erbinPDZ domain (ePDZ). TULIP (Tunable, Light-controlled Interacting Proteins) consists of a long-lived GalDBD-LOVpep and GalAD-ePDZ that upon blue light stimulation heterodimerize, triggering a fivefold increase in expression of β-galactosidase reporter in yeast (Pathak et al., 2014). Of note, C- terminal extensions in TULIP proved instrumental to reduce the otherwise high toxicity, while proper fusions of BD and AD sequences with TULIP modules accounted for the removal of unwanted reporter activation in dark (Pathak et al., 2014).

A newly developed inducible promoter system used the bacterial EL222 transcription factor fused to an NLS and the VP16 AD to direct transcription in eukaryotic cells upon recognition of its cognate DNA sequence, the regulatory element C120. Upon blue light irradiation, the nuclear-targeted helix-turn-helix DNA-binding domain (HTH-DNA binding domain) caged in the dark by the LOV domain, is exposed facilitating dimerization of EL222 (Figure 2B). EL222 dimer then binds to C120 promoter, triggering over a 100-fold transcription of reporter gene in HEK-293T cells. Strikingly, zebrafish embryos injected with small amounts of EL222 mRNA displayed high levels of mCherry reporter under the C120 regulatory element with minimal leakiness in dark and reduced morphological defects and toxicity compared to controls (Motta-Mena et al., 2014). More recently, an updated version of the system, TAEL (TA4-EL222), allowed for more rapid kinetics, higher specificity and lower toxicity than the previous version by substituting the VP16 activation domain for KalTA4. Here, the authors used TAEL to specifically produce ectopic endodermal cells in the presumptive ectoderm of early stage zebrafish embryos, via targeted sox32 induction, as well as to promote gene editing by combining TAEL with the CRSPR/Cas9 system. A notable advantage was the achievement of spatial expression by simply closing the field diaphragm on an epifluorescence microscope (Reade et al., 2017).

##### Light-dependent subcellular localization

Most of the optogenetic tools described until this point are based on the ability of the light-sensor domain to dimerize. An independent strategy proposes the use of a modified monomeric form of AsLOV to cage small localization peptides inside the LOV- domain to shuttle TF between subcellular compartments. By encaging NLS or, alternatively, an NES inside the Jα-helix of the AsLOV monomeric form, a specific TF can accumulate in the nucleus or cytoplasm to trigger or stop transcription upon blue light irradiation. Coincidentally, two independent labs reported a pair of tools to control import (LINuS and LANS) (Niopek et al., 2014; Yumerefendi et al., 2015) (Figure 2C), and export (LEXY and LINX) (Niopek et al., 2016; Yumerefendi et al., 2016) (Figure 2D) of proteins to and from the cell nucleus, respectively. Both designs included the addition of a counter-active signal peptide outside the LOV-cage. This approach assured that the system localized in the appropriate compartment in dark and quickly relocated after light illumination to, then, return to the original state and location when stimulation ceased.

Following this idea, a combination of the import switch LANS with LexA-DBD and Gal4AD led to a 21-fold change in β-galactosidase expression in yeast, demonstrating LANS capability to control the activity of a TF. Further assays in *Caenorhabditis elegans* embryos confirmed rapid and precise spatial translocation of the system upon blue light activation *in vivo* (<2 min) (Yumerefendi et al., 2015). Similarly, LINuS light switch in combination with cyclin B1 and a CDK1 mutant triggered mitosis upon blue light irradiation in HEK293 cells (Niopek et al., 2014). On the other hand, the LINX export system fused to LexA-DBD exhibited up to eight times lower levels of β-galactosidase reporter in yeast when exposed to blue light than those cells kept in dark (Yumerefendi et al., 2016). Then as well, export system LEXY fused to LexA-DBD triggered up to 15-fold increase in firefly luciferase reporter in dark as well as successfully controlled p53 transcriptional activity in H1299 cells (Niopek et al., 2016). These systems proved to be an efficient and fast way to control transcription with an average of 2–10-fold increase under proper conditions. However, all of these shuttle tools showed certain levels of background activity in dark states, probably due to spontaneous protein turn over. To correct the observed leakiness, specific mutations were directed to the LOV domain causing modifications in the lifetime of the open state and reversion of the light-sensor among others (Niopek et al., 2016). These approaches would not only confer more specificity to these optogenetic tools, but also potentially increase the fold ratio observed in the initial reports.

It is important to note that diverse proteins of interest (POI) affect the kinetics of each of these tools differently. Thus, combination of directed mutations in POI specific domains and/or LOV Jα helix or flavin-binding site as well as addition of extra localization signals could better adjust the dynamics of the tool to achieve the desired outcome. Alternatively, combination of these tools with other already existing dimeric LOV2-based systems, such as iLid, can lead to similar results. iLid consists of two improved light-inducible dimers that colocalize under blue light within seconds and revert to dark state in minutes. Hence, by fusing one dimer to the mitochondria and the other to LINX, Khulman’s team significantly reduced activation of β-galactosidase by sequestering the TF out from the nucleus to the mitochondria during the light state (Figure 2E) (Yumerefendi et al., 2016). Alternatively, Hahn’s lab described a new-engineered peptide ZDK, which binds selectively to the dark state of LOV2. Thus, by anchoring either ZDK or LOV2 to the mitochondrial membrane, the TF fused to the other dimer is sequestered away during the dark state. Therefore, this new system, named LOVTRAP, releases the TF from the mitochondria only after blue light irradiation while the light-dependent undocking of the localization signal allows re-localization to the site of action (Figure 2F) (Wang et al., 2016).

To a similar end, Kwon’s lab recently proposed a blue-light-inducible TEV protease system (BLITz) (Lee et al., 2017a). BLITZ consists of two main components: (a) a membrane-tethered CIBN, fused to the N-terminal region of TEV protease (TEV-N), the AsLOV2 caging a protease–cleavage sequence (TEVseq), and the TetR-VP16 AD; and (b) a fusion protein of CRY2PHR with C-terminal region of TEV protease (TEV-C). Upon blue light irradiation, CRY2PHR-TEV-C and CIBN-TEV-N heterodimerize restoring TEV protease function (Wehr et al., 2006). Simultaneously, blue light causes the undocking of TEVseq from AsLOV2. Then, cleavage of TEVseq by TEV protease leads to release of TetR-VP16 AD triggering a 20-fold increase of reporter expression in HEK293 cells with minimal background and precise temporal resolution (Lee et al., 2017a). In addition, the authors combined BLITZ with iTango, a system that relies on specific binding of a ligand to dopamine receptor 2 (DRD2) to recruit TEV-C (Barnea et al., 2008; Lee et al., 2017a). Thus, blue light causes exposure of TEVseq, which is recognized and cleaved by the reconstituted TEV protease. This releases TetR-VP16 AD to activate expression of reporter genes in neurons both *in vitro* and *in vivo* (Lee et al., 2017a).

Shortly after, the same team proposed another innovative system referred to as Cal-Light. This system allows manipulating gene expression in specific subgroups of cells in cultured neurons and brain slices in the presence of blue light and calcium (Lee et al., 2017b). In Cal-Light, TEV- N, -TEVseq, and TetR-VP16 are fused to a membrane-tethered calcium sensor (CaM), while TEV-C is fused to calcium sensor M13. When calcium levels increase in the cytosol, M13 binds to CaM, reconstituting TEV protease function. Then, when irradiated with blue light, TEVseq becomes available to the reconstituted TEV protease, releasing the TF and then promoting gene expression (Lee et al., 2017b).

### Toward the Dark Side

#### UVR8

**FIGURE 3:**
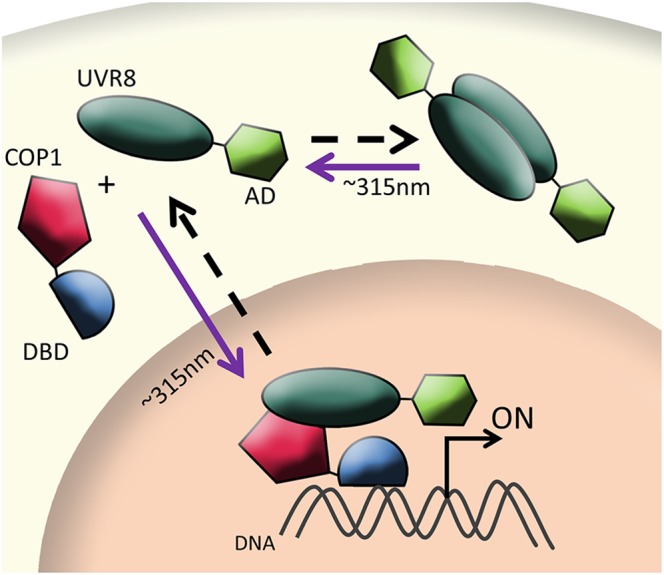
Schematic illustration of UV-dependent system (UVR8-COP1). In darkness, UVR8 accumulates in cytoplasm as homodimers. Upon UV-B light irradiation, UVR8 monomerizes and associates with its binding partner COP1 to reconstitute a split transcription factor (DBD and AD). Then, the complex translocates to the nucleus and promotes gene expression. Arrows depict direction of activity and light conditions: the purple arrow (∼315 nm) represents UV-B light-dependent activation, while the dashed black arrow represents darkness or absence of light stimuli.

UVR8 (280–315 nm) is a photo sensing protein able to detect ultraviolet-B light allowing plants to adapt to the environment during conditions of stress as well as control seedling, leaf development, photomorphogenesis, and growth, among others. UVR8 consists of a seven bladed β-propeller type domain rich in tryptophan (Trp) which homodimerizes in darkness. Contrary to previously discussed photoreceptors, UVR8 does not require addition of a chromophore to undergo conformational change upon light stimulation. Instead, when irradiated with UV-B light, the Trp residues, acting as chromophore, cause the receptor to monomerize. In this form, UVR8 can interact with the WD-40 domain of the binding cofactor Constitutively Photomorphogenic 1 (COP1) and accumulates in the nuclei of the cells (Figure 3) (Rizzini et al., 2011). Mutations in UVR8 Trp residues or COP1 WD-40 domain resulted in failure to respond to UV-B light or interaction between dimers, respectively. Then, similar to other optogenetic two-hybrid systems, by fusing UVR8 to the transcription activation domain NF-κB, and Gal4-DBD to COP1, co-transfected U2OS cells showed up to 50-fold change in luciferase expression upon UV-B light irradiation (Crefcoeur et al., 2013).

The ability to work without a chromophore makes the adaptation to animal models potentially easier than in the case of the PhyB/PIF system. Moreover, the poor response of UVR8 to wavelengths comprised on the visible spectrum makes this tool a good candidate to combine with other of the existing optogenetic tools. However, similar to the limitations observed in blue light-dependent systems, the short wavelength needed for activation is probably UVR8’s major drawback. UV-B offers low penetrance in tissue with an even greater risk of photo toxicity than blue light.

## The Systems Dilemma

Optogenetics has given rise to a new set of tools that allows for spatiotemporal control of gene expression with unprecedented resolution. However, differences in chromophore properties, light wavelengths and conditions, as well as system kinetics and reversibility create room for questions about which system is more appropriate for a particular animal model or interest (Pathak et al., 2014; Tucker et al., 2014; Di Ventura and Kuhlman, 2016; Hallett et al., 2016). In addition, there are issues only encountered after experimentation in a specific biological context or dependent on the POI. Therefore, we provided here an updated, comparable and helpful description of all properties and challenges of the available optogenetic expression systems. This will certainly help the audience to choose the best option to manipulate transcription in a light-dependent manner. For instance, when thinking to implement a two-hybrid approach in multicellular organisms, the PhyB-PIF system appears as one of the most suitable options. Long periods of light irradiation can turn toxic for the organism of study; however, PhyB possesses a long half-life allowing for shorter periods of light pulses to achieve similar or even greater expression values than other tools. Furthermore, the required red light has a better penetrance in tissue and presents a less toxic effect than blue light irradiation. In addition, it is the only tool among the ones described that possesses an off-switch wavelength. Nonetheless, phytochromes depend upon the external administration of PCB to the organism. In unicellular models, PCB is easily provided through the media. However, PCB administration to more complex organisms offers an extra unique set of challenges. As previously mentioned, cells could be engineered to produce PCB from a heme group. This approach, however, involves a series of genetic modifications. Firstly, the model of choice requires independent engineering to express the necessary enzymes to synthesize PCB. More troubling is the need to knock down all potential endogenous proteases that could compromise the amount of effective enzyme available. Subsequent secondary effects of this approach are still unknown and could potentially interfere not only with the pathway of interest but also with the normal life of the individual.

A two-hybrid blue light-based system or any of the translocation tools, on the other hand, could be a fitting alternative. Blue-light dependent systems require FMN –a molecule present in all living cells – bypassing the need of any external supplements. Although, blue light systems lack of an off-switch light, they have short half-lives, allowing for quick reversion back to the dark state. Nonetheless, strong and prolonged gene expression calls for longer periods of blue light irradiation, which is known to interfere with signaling transduction, circadian pathways and transcription activity, to name some. For example, TAEL activation led to a delay in development of zebrafish embryos upon activation, although no long-term effects or abnormalities were later observed (Reade et al., 2017). Applying light pulses that alternate short on-off periods of light can be a good strategy for these blue-dependent opto-tools. Tucker et al. (2014) showed that 1s pulses were capable of producing near maximum stimulation with no significant toxicity in cells. Yet, further experimentation might be needed in a specific model of interest to achieve the ideal on-off interval for optimal activation.

Leakiness of the systems as well as the possibility of spontaneous activation of expression in dark conditions is the most prevalent complication among first generation optogenetic systems. Point mutations that stabilize the photo sensing protein and slow its kinetics can improve the outcome by weakening the dark state binding. Coupling preferences between heterodimer optogenetic tools and split domains of TF can, instead, lead to different degrees of background activity. For instance, fusion between CIB1 and DBD can trigger transcription independently of CRY2 association since CIB1 functions as transcriptional activator on its own (Liu et al., 2012; Konermann et al., 2013). Even switching N- or C- terminal fusion to the POI can result in different background activity or promote unexpected behavior of the tool (Pathak et al., 2014). For example, addition of C-terminal extensions in TULIP constructs led to a significant decrease in toxicity (Strickland et al., 2012; Pathak et al., 2014). Length of the photo sensory gene is also instrumental on the protein behavior. For instance, although functional in cell models, PhyB full length was unsuccessfully expressed in zebrafish embryos. However, a shorter version of PhyB where PAS and histidine kinase related domains were removed, was robustly expressed *in vivo* (Buckley et al., 2016). Moreover, truncated CRY2 (CRY2PHR) resulted in significantly reduced background activity for activation of the MAP kinase pathway compared to full-length CRY2 (Pathak et al., 2014). However, full-length CRY2 performed better in transcriptional studies.

But these differences among tools also offer an exciting new opportunity. Now, not only is spatiotemporal activation easily achievable, but also two or more target genes can be differentially activated with the combination of different optogenetic tools of different wavelengths and/or other inducible systems. For instance, by using short blue/UV light pulses in a background of far red light, Muller et al. (2013b) successfully activated UVR8 while repressing PhyB response. Accordingly, the authors also activated specific PhyB/PIF dependent transcription with undetectable UVR8-dependent expression (Muller et al., 2013b).

## Concluding Remarks

Creation and implementation of optogenetic tools over the past several years has changed the way we think about regulation of gene expression and protein activity. The unprecedented spatiotemporal control acquired through these revolutionary tools promises a better insight on mechanisms involved not only in biological processes but also those associated with a range of complex human disorders. However, each and every set of optogenetic tools presents a unique group of features, advantages and challenges to consider depending on our research interest. While the major advantage of PhyB-dependent tools resides in the existence of two wavelengths to provide a rapid and stable on/off switch; the FAD and FMN endogenous cofactor associated with CRY2-based and LOV-dependent systems allow for an easier translation and adaptation of light dependent tools to animal models. Thus, thoughtful considerations into activating wavelengths, chromophore requirements, dimerization properties, or reversibility are fundamental to successfully achieve photo regulation in a model of interest. Nevertheless, it is clear that bringing light to transcription will illuminate many avenues of research for years to come.

## Author Contributions

LDM and DER-L conceived the manuscript. LDM and PR drafted the manuscript. LDM prepared the figures. DER-L coordinated all sections and edited the article. All authors read and approved the final version of the manuscript.

## Conflict of Interest Statement

The authors declare that the research was conducted in the absence of any commercial or financial relationships that could be construed as a potential conflict of interest.
